# Antidepressant Drugs Transactivate TrkB Neurotrophin Receptors in the Adult Rodent Brain Independently of BDNF and Monoamine Transporter Blockade

**DOI:** 10.1371/journal.pone.0020567

**Published:** 2011-06-07

**Authors:** Tomi Rantamäki, Liisa Vesa, Hanna Antila, Antonio Di Lieto, Päivi Tammela, Angelika Schmitt, Klaus-Peter Lesch, Maribel Rios, Eero Castrén

**Affiliations:** 1 Sigrid Jusélius Laboratory, Neuroscience Center, University of Helsinki, Helsinki, Finland; 2 Centre for Drug Research, Faculty of Pharmacy, University of Helsinki, Helsinki, Finland; 3 Molecular Psychiatry, Laboratory of Translational Neuroscience, Department of Psychiatry, Psychosomatics and Psychotherapy, University of Würzburg, Würzburg, Germany; 4 Department of Neuroscience, Tufts University School of Medicine, Boston, Massachusetts, United States of America; Sapienza University of Rome, Italy

## Abstract

**Background:**

Antidepressant drugs (ADs) have been shown to activate BDNF (brain-derived neurotrophic factor) receptor TrkB in the rodent brain but the mechanism underlying this phenomenon remains unclear. ADs act as monoamine reuptake inhibitors and after prolonged treatments regulate brain *bdnf* mRNA levels indicating that monoamine-BDNF signaling regulate AD-induced TrkB activation *in vivo*. However, recent findings demonstrate that Trk receptors can be transactivated independently of their neurotrophin ligands.

**Methodology:**

In this study we examined the role of BDNF, TrkB kinase activity and monoamine reuptake in the AD-induced TrkB activation *in vivo* and *in vitro* by employing several transgenic mouse models, cultured neurons and TrkB-expressing cell lines.

**Principal Findings:**

Using a chemical-genetic TrkB^F616A^ mutant and TrkB overexpressing mice, we demonstrate that ADs specifically activate both the maturely and immaturely glycosylated forms of TrkB receptors in the brain in a TrkB kinase dependent manner. However, the tricyclic AD imipramine readily induced the phosphorylation of TrkB receptors in conditional *bdnf* ^−/−^ knock-out mice (132.4±8.5% of control; P = 0.01), indicating that BDNF is not required for the TrkB activation. Moreover, using serotonin transporter (SERT) deficient mice and chemical lesions of monoaminergic neurons we show that neither a functional SERT nor monoamines are required for the TrkB phosphorylation response induced by the serotonin selective reuptake inhibitors fluoxetine or citalopram, or norepinephrine selective reuptake inhibitor reboxetine. However, neither ADs nor monoamine transmitters activated TrkB in cultured neurons or cell lines expressing TrkB receptors, arguing that ADs do not directly bind to TrkB.

**Conclusions:**

The present findings suggest that ADs transactivate brain TrkB receptors independently of BDNF and monoamine reuptake blockade and emphasize the need of an intact tissue context for the ability of ADs to induce TrkB activity in brain.

## Introduction

TrkB (tropomyosin-related kinase B) neurotrophin receptor transduces intracellular signaling events that are critical for neuronal differentiation, survival and plasticity throughout life [1]–[5]. Brain-derived neurotrophic factor (BDNF) is the main endogenous ligand for TrkB [3], but recent evidence demonstrates that TrkB can also be transactivated independently of BDNF or other neurotrophins through neuromodulator receptors [6], [7] and small molecules [8], [9].

Abnormal TrkB receptor signaling has been linked to a number of central nervous system (CNS) diseases such as mood and memory disorders and addiction [10]–[12]. Accumulating evidence suggests that antidepressant drugs (AD) that regulate the brain levels of monoamine neurotransmitters serotonin and norepinephrine, act at least partially by activating TrkB receptor signaling in brain [13], [14]. ADs have been shown to rapidly induce the phosphorylation and activation of TrkB receptors in the rodent cortex and hippocampus [13], [15]. When administered chronically, ADs also increase BDNF mRNA and protein levels and TrkB phosphorylation in brain [13], [15], [16]. Furthermore, animal studies suggest that many of the behavioral and functional actions of ADs are attenuated in mice with reduced BDNF signaling in brain [13], [17]. Levels of BDNF are reduced in the brain and serum of depressed patients and the levels are returned back to normal range upon a successful treatment with ADs [18], [19].

In this study we have examined several potential molecular mechanisms of AD-induced TrkB activation *in vitro* and *in vivo*. We show that both mature and immature forms of TrkB can be specifically tyrosine phosphorylated by ADs, but neither the endogenous ligand BDNF, nor the serotonin transporter (SERT), the principal target of many ADs, is required for this effect. However, the observation that ADs or serotonin (5-HT) or norepinephrine (NE) do not activate TrkB phosphorylation *in vitro* argues that ADs do not directly bind to TrkB receptors.

## Results

### Antidepressant drugs specifically activate TrkB receptors in mouse brain

**Figure 1:**
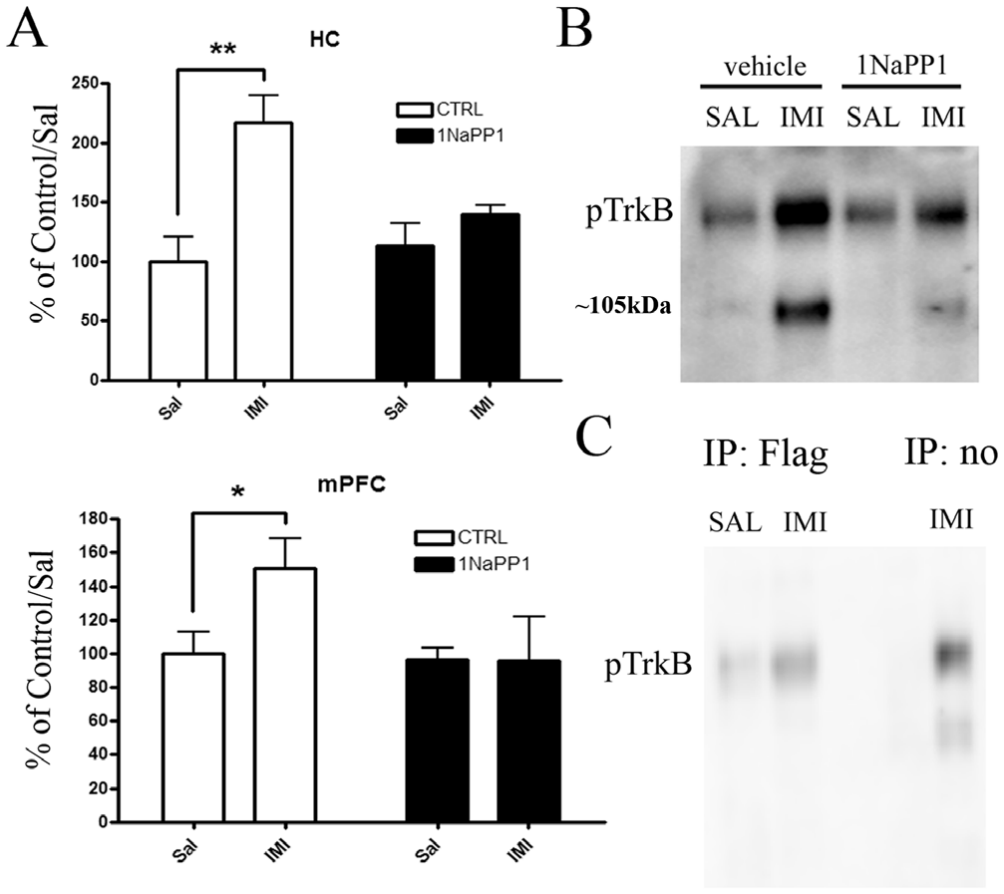
Imipramine specifically activates TrkB receptors in the mouse brain. **A**) The ability of imipramine (30 mg/kg, i.p., 30 min; n = 6/group) to induce rapid TrkB phosphorylation in the hippocampus and medial prefrontal cortex of TrkB^F616A^ mutant mice is abolished with 1NaPP1 pretreatment (25 µM for 1-week in drinking water+83 ng/g co-injection with imipramine). **B**) A representative blot showing TrkB kinase dependent action of imipramine-induced phosphorylation of TrkB (Y816) and the ∼105 kDa protein. **C**) A representative blot showing imipramine-induced TrkB phosphorylation in flag-precipitated pool of protein from the brains of mice over-expressing flag-tagged catalytic TrkB receptors. Data is presented as percentage of control/saline ± standard error of mean (SEM). *<0.05, **<0.01; two-way ANOVA with Newmann-Keuls *post hoc* test.

Previous studies suggest that BDNF-TrkB signaling is critical for the behavioral effects of ADs [13], [17] and that ADs activate Trk receptors *in vivo*. We have previously shown that several independent antibodies raised against phosphorylated tyrosines Y705/706 or Y816 within the intracellular domain of TrkB all show increased phospho-TrkB levels after acute and chronic AD treatment [13], [15], while no increase is detected with antibodies against the shc binding site at pY515. Furthermore, immunoprecipitation with Trk specific antibodies and probing with pTyr-antibodies also reveals increased TrkB phosphorylation [13], [15], [20]. However, since phospho-Trk antibodies are not completely specific for TrkB, we investigated whether the protein phosphorylated by the acute AD treatment is indeed TrkB. We pretreated TrkB^F616A^ knock-in mice [21] with NaPP1, a chemical that specifically inhibits TrkB kinase activity in these mutant mice, and then injected the mice acutely with imipramine. Whereas imipramine readily induced rapid activation of brain TrkB in vehicle-treated TrkB^F616A^ knock-in mice, NaPP1 treatment abolished this effect (**Figure 1A–B**). Furthermore, when we treated transgenic mice over-expressing *flag*-tagged TrkB receptors in adult neurons (TrkB.TK+) [22], [23] with imipramine, we observed enhanced phosphorylation of the TrkB specific band when compared to wild-type mice (data not shown). Importantly, when TrkB receptors were immunoprecipitated with a Flag antibody from TrkB.TK+ mouse brain homogenates, phospho-TrkB signal was more intense in samples of imipramine treated animals (**Figure 1C**). Collectively, these data demonstrate that imipramine specifically induce phosphorylation of TrkB receptors in mouse brain.

### The immaturely glycosylated form of TrkB is phosphorylated by antidepressants

**Figure 2:**
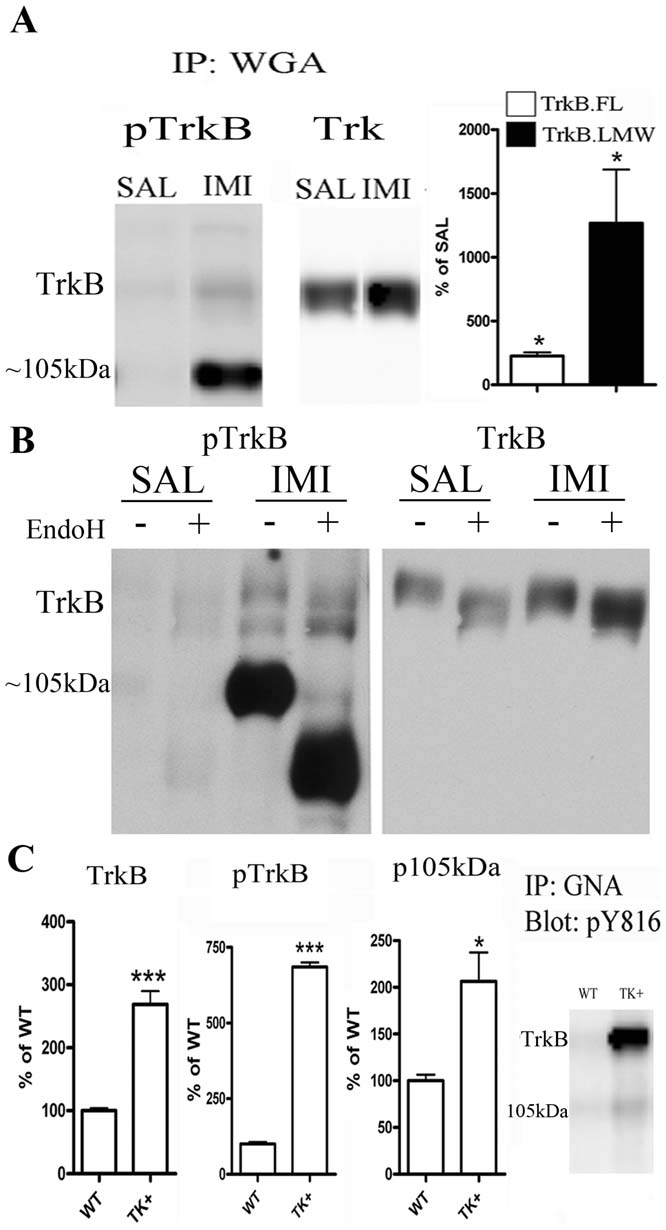
Antidepressant drugs activate the immaturely glycosylated form of TrkB. **A**) Acute imipramine treatment induces the phosphorylation (Y816) of full-length and low-molecular weight (LMW; ∼105 kDa) TrkB receptors in mouse brain. n = 6/group. **B**) Antidepressant-induced ∼105 kDa protein is sensitive to Endo-H digestion. A representative blot of triplicate data. **C**) Total TrkB, phosphorylated TrkB (Y816) and phosphorylated ∼105 kDa protein levels are increased in the brains of mice over-expressing catalytic TrkB receptors. n = 5/group. Data is presented as percentage of control ± standard error of mean (SEM). *<0.05, ***0.005; unpaired two-tailed t-test.

As we have previously shown [13], an additional low-molecular weight (LMW) phospho-Trk –immunoreactive protein (about 105 kDa) is robustly phosphorylated in the rodent brain after single or repeated AD treatment (**Figure 2A**). This phosphorylated protein is detected by the same antibodies that demonstrate the phosphorylation of TrkB after AD treatment (**Figure S1A–B**) and has been detected following TrkB immunoprecipitation and hybridization to pTyr antibodies [13], [20]. AD-induced phosphorylation of both the full-length TrkB and the 105 kDa protein is also readily detected in different brain regions including striatum, midbrain and cerebellum (data not shown), but, similar to full-length TrkB, its phosphorylation is diluted in whole brain homogenate (**Figure S1C**). However, this band cannot be reliably detected by antibodies against the non-phosphorylated intracellular domain of Trk receptors (**Figure 2A**).

This lower molecular weight protein might represent an immaturely glycosylated form of catalytic TrkB [24], as TrkB transactivation has been shown to coincide with accumulation of intracellular immaturely glycosylated TrkB species [6], [7], [25]. We therefore further examined the glycosylation structure of this protein using endoglycosidase-H (Endo-H) that cleaves immature high-mannose rich *N*-glycans out of proteins. Endo-H digestion produced a slight reduction in the molecule weight of the mature full-length TrkB, suggesting that the mature TrkB still contains immature-type glycan residues (**Figure 2B**), as also observed before for TrkA [26]. Importantly, Endo-H treatment strongly reduced the molecular weight of 105 kDa protein (**Figure 2B**), suggesting that essentially all the glycan residues in this protein represent immature high-mannose rich *N*-glycans. These data are consistent with the interpretation that the 105 kDa protein represents an immaturely glycosylated and intracellularly located species of TrkB. This interpretation is further supported by the observations that the basal phosphorylation levels of this phosphoprotein are increased in the brains of TrkB over-expressing mice (**Figure 2C**) and that the activation of this band is lost after 1NaPP1 treatment in the TrkB^F616A^ mice (**Figure 1B**).

### Antidepressant-induced TrkB activation does not require BDNF

Previous studies have shown that acute AD treatment does not influence BDNF mRNA or protein levels [13], [16]. Since it has recently been suggested that pro and mature forms of BDNF might have different capacities to activate TrkB [27], we investigated the effects of acute antidepressant treatment on proBDNF cleavage in brain. However, we were not able to detect any proBDNF signal in wild-type mouse brain, even if the antibody readily detected the recombinant proBDNF control protein (**Figure S2A**). Nevertheless, acute fluoxetine treatment, with a dose and time point (30 mg/kg; 1 hour) which induced TrkB phosphorylation in mouse hippocampus [13], [15], failed to produce any significant changes in the levels of the mature BDNF (mBDNF) in mouse brain as detected with western blotting (**Figure S2A**). Furthermore, fluoxetine did not influence the activity of tissue plasminogen activator (tPA), the major regulator of pro-BDNF cleavage into mBDNF (**Figure S2B**). These data suggest that ADs do acutely not influence BDNF levels or processing in brain.

**Figure 3:**
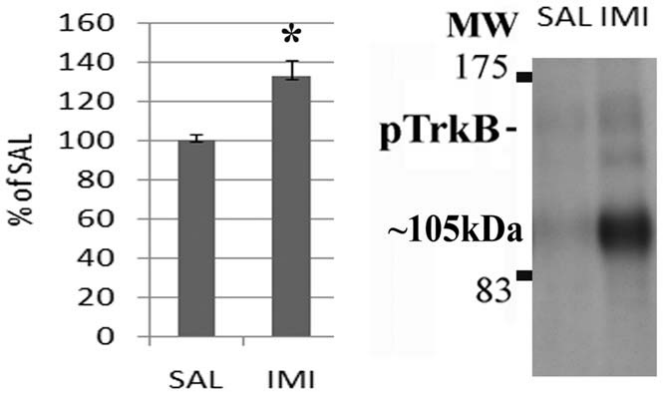
Role of BDNF in antidepressant-induced rapid TrkB activation in brain. Imipramine (30 mg/kg, 30 min, i.p.) readily increases the phosphorylation of TrkB receptors (Y816) in forebrain specific BDNF^−/−^ knock-out mice (BDNF^2L/2LCk-cre^) n = 4/group. Data is presented as percentage of control ± standard error of mean (SEM). *<0.05; unpaired two-tailed t-test.

Although BDNF is the main ligand of TrkB, recent evidence suggests that TrkB can also be activated independently of BDNF in neurons [6], [8], [9]. We therefore used conditional BDNF mutant mice (BDNF^2L/2LCk-cre^) lacking BDNF in forebrain regions [28] to investigate whether BDNF is required for the AD-induced TrkB activation in brain *in vivo*. Imipramine readily induced tyrosine phosphorylation of both the mature and the immature glycosylated forms of TrkB in the hippocampus of conditional BDNF^2L/2LCk-cre^ mice (**Figure 3**). Similarly, imipramine produced an increase in brain TrkB phosphorylation in heterozygous *bdnf* ^+/−^ null mice and wild-type mice (data not shown). These data demonstrate that ADs activate TrkB receptors in the mouse brain in a manner independent of BDNF.

Adenosine has been shown to transactivate TrkB receptors *via* adenosine-_2A_ signaling in the absence of BDNF *in vitro* and *in vivo* [6], [29] and to enhance TrkB signaling [30]. Furthermore, some ADs have been shown to acutely increase the extracellular levels of adenosine by reducing adenosine reuptake [31]. We therefore tested whether prior pharmacological inhibition of adenosine A_2A_ receptors with ZM241385 might block the acute effects of ADs on TrkB phosphorylation. We found that, imipramine increased the phosphorylation of TrkB receptors similarly in mice pretreated with saline or active dose [32], [33] of ZM241385 (**Figure S3**), suggesting that A_2A_ receptors were not involved.

**Figure 4:**
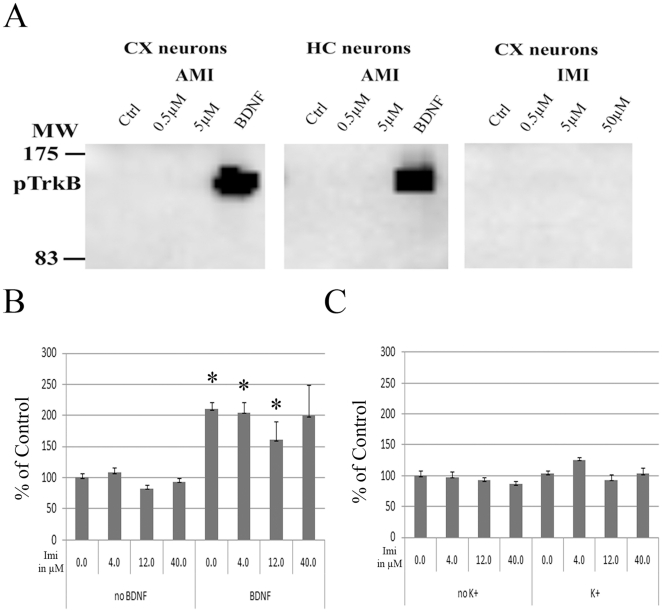
Antidepressant drugs amitriptyline and imipramine do not regulate TrkB phosphorylation in primary neurons. **A**) Whereas BDNF (20 ng/ml; 15 min) robustly increases the phosphorylation of TrkB (Y816) in E18 rat cortical and hippocampal neurons (14 DIV), amitriptyline (left & middle; 0.5 µM, 5 µM; 15 min) and imipramine (0.5 µM, 5 µM; 50 µM; 15 min) produces no change on TrkB phosphorylation. Representative blot of triplicate data. **B**) Imipramine pre-treatment (4, 12, 40 µM; 15 min) did not facilitate BDNF-induced (5 ng/ml; 15 min) TrkB phosphorylation in E18 rat cortical neurons as measured with phospho-TrkB ELISA. n = 4/group. **C)** Imipramine pre-treatment (4, 12, 40 µM; 15 min) did not regulate TrkB phosphorylation in its own or in combination with depolarization stimuli (50 mM KCl; 15 min) as measured with phospho-TrkB ELISA. n = 4/group. Data is presented as percentage of control ± standard error of mean (SEM). *<0.05; one-way ANOVA with Newmann-Keuls *post hoc* test.

Amitriptyline, but not imipramine, was recently shown to directly bind and transactivate TrkB receptors *in vitro* [9]. We therefore tested whether amitriptyline, imipramine or other selected drugs could directly phosphorylate TrkB receptors in two different cell models: fibroblast expressing catalytic TrkB receptors and E18 rat primary hippocampal and cortical neuronal cultures. In both of these cells, BDNF produces a robust phosphorylation of TrkB. However, exposure to tested ADs, including amitriptyline, or other tested drugs did not regulate TrkB phosphorylation status in these cultures (**Figures 4A**, **S4**). We further tested whether ADs might potentiate the pTrkB response induced by a small dose of BDNF or whether depolarization of neurons might render them sensitive to ADs *in vitro*. Imipramine did not facilitate BDNF-induced TrkB phosphorylation *in vitro* (**Figure 4B**), which is in line with the findings in BDNF deficient mice. Similarly, even when ADs were coupled with depolarization stimuli (50 mM K^+^), no significant changes in TrkB phosphorylation were seen (**Figure 4C**).

### TrkB activation by antidepressant drugs is not mediated by the serotonin transporter or monoamine transmitters

**Figure 5:**
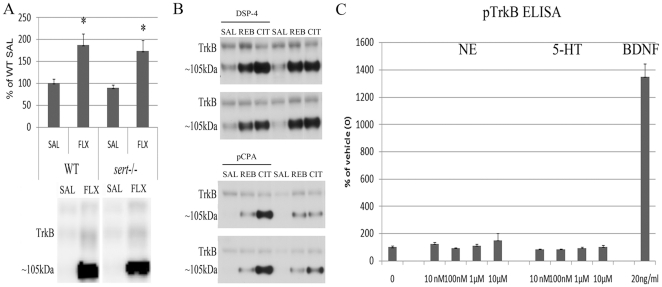
Monoamines and monoamine reuptake in TrkB activation *in vitro* and *in vivo*. **A**) Serotonin selective reuptake inhibitor fluoxetine produced essentially similar changes on hippocampal TrkB phosphorylation in the brains of wild-type and serotonin transporter KO mice, *sert* ^−/−^. n = 3–5/group. **B**) Whereas the ability of serotonergic antidepressant citalopram (20 mg/kg, i.p., 60 min) and norepinephrinergic antidepressant reboxetine (20 mg/kg, i.p., 30 min) to induce full-length TrkB receptor phosphorylation (Y816) in mice depleted of serotonin (with *p*CPA) or norepinephrine (with DSP-4) are reduced, ∼105 kDa protein is heavily phosphorylated by both drugs in the hippocampi of these mice. Representative blots. n = 6–7/group. **C**) Norepinephrine (NE; 10 nM-10 µM; 15 min) and serotonin (5-HT; 10 nM-10 µM; 15 min) produced no changes on TrkB phosphorylation in BDNF-responsive E18 rat cortical neurons (15DIV) as measured with phospho-TrkB ELISA. n = 4/group. Data is presented as percentage of control ± standard error of mean (SEM). *<0.05; two-way ANOVA with Newmann-Keuls *post hoc* test.

Essentially all clinically used antidepressant drugs acutely increase the extracellular levels of NE and/or 5-HT in brain and we therefore investigated the role of these monoamines in the AD-induced TrkB transactivation *in vivo* and *in vitro*. First we examined the effect of fluoxetine, a prototypic SERT selective reuptake inhibitor (SSRI), on TrkB receptor phosphorylation in SERT knockout mice (*sert* ^−/−^). A 6–10 fold up-regulation of extracellular 5-HT levels in *sert* ^−/−^ mice [34] did not regulate basal TrkB phosphorylation levels in hippocampus when compared to the wild-type controls (89.43%±6.43% of wild-type, P = 0.19, Student t-test). Importantly, fluoxetine readily induced the phosphorylation of both the mature and immature forms of TrkB in the brains of *sert* ^−/−^ mice in a manner indistinguishable of the wild-type mice (**Figure 5A**), indicating that SERT is dispensable to the fluoxetine-induced TrkB autophosphorylation.

Because the selectivity of the ADs against different transporters is only relative, we performed chemical lesion experiments to reduce brain 5-HT (by *p*CPA treatment) and NE levels (by DSP4) and used selective 5-HT and NE transporter blockers citalopram and reboxetine, respectively. As noted before [15], citalopram and reboxetine produced a non-significant trend of increase in TrkB autophosphorylation levels in *p*CPA and DSP4 treated mice, respectively (**Figure 5B**). However, citalopram and reboxetine induced a strong and highly significant increase in the phosphorylation of the immaturely glycosylated form of TrkB in *p*CPA and DSP4 treated mice, respectively (**Figure 5B**). These observations suggest that even when brain 5-HT and NE levels are very low, ADs can activate at least the immaturely glycosylated form of TrkB.

Finally, we tested whether NE or 5-HT would directly regulate TrkB phosphorylation in cultured primary neurons. Under conditions where BDNF robustly induced TrkB phosphorylation, incubation with different concentrations of NE or 5-HT did not regulate TrkB phosphorylation levels in primary neuronal cultures (**Figure 5C**). Collectively, these data suggest that TrkB receptor is activated in mouse brain by ADs independent of monoamine reuptake inhibition.

## Discussion

Emerging evidence suggests a key role of the BDNF-TrkB signaling in the regulation of many of the molecular and behavioral actions of ADs. ADs acutely and chronically increase TrkB signaling [13], [15]. Moreover, chronic, but not acute, AD treatment increases BDNF synthesis in the rodent brain [16], [35]. BDNF injection and TrkB activation produce AD-like responses in rodents [36]–[38], while mice deficient of BDNF or with inhibited TrkB signaling do not respond to ADs in the forced swim test [13], [17], the classical paradigm for AD effectiveness. These data suggest that, at least in rodents, activation of TrkB receptors induced by BDNF is essential for the antidepressant effect. However, we show here that rapid activation of TrkB in response to AD administration *in vivo* does not require BDNF release. This finding does not rule out the role of BDNF in regulating TrkB activation following chronic AD treatment. Since acute AD treatment increases phosphorylation of CREB, a critical upstream regulator of BDNF synthesis in a TrkB dependent manner [13], it is tempting to speculate that this ligand-independent TrkB activation is contributing the AD-induced BDNF synthesis in brain [39] which further leads to BDNF-dependent TrkB phosphorylation after prolonged AD administration.

Fluoxetine and SSRIs act primarily by blocking 5-HT reuptake in brain and BDNF, through TrkB, is a crucial regulator of serotonergic innervation [40], [41]. However, neither the SERT nor the monoamines 5-HT or NE appear to be required for the activation or TrkB by the ADs. We have previously shown that representatives of all the different chemical classes of ADs similarly increase TrkB phosphorylation in mouse brain, suggesting that the monoamine independent TrkB activation may be a common feature for all the ADs. Accumulating evidence has shown that ADs, including fluoxetine and tricyclic ADs, have several additional targets in cells such as neurotransmitter receptors [42], [43], ion channels [44], Sigma-1 receptors [45] and adenosine reuptake proteins [31] that could potentially be involved in regulating TrkB signaling. Since adenosine-A_2A_ receptor signaling has been linked to TrkB signaling [30], we tested the role of this receptor in AD-induced TrkB response by pharmacologically blocking this receptor before imipramine challenge. However, no change was observed compared to control treatment indicating that adenosine is not a critical regulator of TrkB activation in response to AD treatments.

While all the different ADs readily induce TrkB autophosphorylation in rodent brain [13], [15], neither these compounds nor monoamines do, in our hands, induce TrkB phosphorylation *in vitro* in cultured cortical or hippocampal neurons, or in cell lines stably expressing TrkB receptors. A recent study reported that amitriptyline, but not imipramine, binds to TrkB receptors, induces their dimerization and autophosphorylation in cultured hippocampal neurons [9]. The reasons that underlie the discrepancy between that study and our results with amitriptyline are currently unclear, however, they may be related to the culture conditions used. The lack of activation of TrkB by ADs in cultured neurons is in line with our recent unpublished observations showing that the ability of ADs to activate TrkB *in vivo* is developmentally regulated: ADs do not activate TrkB in embryonic or early postnatal mice, but the ability of these compounds to induce pTrkB response appears only around postnatal day 15 (P15) (ADL, TR and EC, submitted). Taken together, these data suggests us that ADs do not directly bind to TrkB, but, instead, emphasize the importance of developmental processes and intact tissue context in the ability of small molecule weight drugs such as ADs to activate TrkB autophosphorylation *in vivo*.

## Materials and Methods

Animal experiments - Wild-type (C57/BL6), TrkB.TK+ mutant [22], [23], *trkB* ^F616A^ knock-in [21], BDNF^2L/2LCk-cre^ [28] and *sert* ^−/−^ knock-out mice [46], [47] were used in animal experiments. Mice were group-housed in standard laboratory conditions and food and water were freely available. All the experiments were carried out according to the guidelines of the Society for Neuroscience and were specifically approved by the University of Helsinki Committee on Animal Experiments (permit: HY 137-05) or the County Administrative Board of Southern Finland (Permit: ESLH-2007-09085/Ym-23). Unless otherwise stated all the tested chemicals used in these studies are purchased from Sigma-Aldrich.

*In vivo* drug treatments - Mice received a single i.p. injection of tested chemical or saline and after indicated time the mice were killed with CO_2_ and brain area of interest dissected on a cooled plastic dish. Next the samples were lyzed in NP++ buffer (137 mM NaCl, 20 mM Tris, 1% NP-40, 10% glycerol, 48 mM NaF, H_2_O, 2× Complete inhibitor mix (Roche) and 2 mM Na_3_VO_4_), incubated on ice (>30 min) and centrifugated (+4°C for 15 min, 16100 *g*). The supernatant was processed further as described below. The following chemicals were used: fluoxetine-HCl (Orion Pharma), citalopram-HBr (GlaxoSmithKline; GSK), imipramine-HCl, moclobemide (kind gift from F Hoffmann-La Roche Ltd), clomipramine-HCl, amitriptyline-HCl, reboxetine (GSK). Reboxetine and moclobemide were first stock-dissolved in DMSO, citalopram in ethanol, others directly in saline. In order to inhibit TrkB kinase activity prior drug administration in TrkB^F616A^ knock-in mice, mice were pre-treated with 25 µM of 1NaPP1 (kindly provided by Prof. Jari Yli-Kauhaluoma, Univ. Helsinki, Finland) for 7 days (in drinking solution) and further co-injected i.p. (83 ng/g) with imipramine or vehicle. ZM241358 was injected i.p. to block adenosine A_2A_ receptors 30 min prior imipramine treatment [33]. Brain NE and 5-HT levels were depleted using DSP-4 (brain NE levels <20% of control; P<0.01, t-test) and *p*CPA (brain 5-HT levels <15% of control; P<0.005, t-test) injections as described previously [15].

Tissue plasminogen activator (tPA) SDS-PAGE zymography - For tPA activity assay, freshly dissected brain samples were homogenized into buffer consisting of 0.1 M Tris-HCl (pH 8.0), 2.5% Triton-X-100, 10 µM leupeptin, 10 µg/ml aprotinin, 1 mM phenylmethanesulfonylfluoride (PMSF). Samples and controls (human recombinant tPA) were loaded under non-reducing conditions in SDS-PAGE containing±human plasminogen (Sigma-Aldrich) and pre-heated non-fat dry milk at low current (∼15–20 mA) over night (O/N) at cold bath. Next the gels were rinsed thoroughly with 2.5% Triton X-100 to remove SDS and allow proteins to renaturate. Next the gels were rinsed thoroughly with 10 mM CaCl_2_ 50 mM Tris-HCl (pH 7.6) to remove Triton X-100 and the caseinolysis was allowed to occur by incubating the gels at +37°C for 16–24 h in the same solution. Caseinolytic areas were shown as translucent areas when the gels were stained with Coomassie Brilliant Blue.

*In vitro* experiments - For the primary neuronal cultures, hippocampi or cortex was dissected from E18 rat embryos and the tissue dissociated in a papain solution (in mg: 10 DL-Cystein-HCl, 10 bovine serum albumin (BSA), 250 glucose, ad 50 ml PBS; 10 min, 37°C). Next the cells were triturated and suspended in a medium containing 9.8 ml of Ca^2+^/Mg^2+^ free HBBS, 1 mM sodium pyruvate, 10 mM HEPES and 10 µl DNAse I. The cells were plated onto poly-*L*-lysine coated 12–24 well culture plates at a cell density of 0.5×10^6^ ml^−1^ (hippocampal) or 1×10^6^ ml^−1^ (cortical). Cells were maintained in neurobasal medium (+2% B27 supplement, 1% penicillin/streptomycin, 1% glutamine and 25 µM glutamic acid; 5% CO_2_, +37°C) for 14 to 15 days *in vitro* (DIV) before treatments. Parental MG87 and MG87-*trkB* fibroblasts [48] were cultured in 12–48 well culture plates in Dulbecco's Modified Eagle's Medium (DMEM) (+10% fetal calf serum, 1% PEST, 1% L-Glutamine, 400 µg/ml G418; 5% CO_2_, +37°C) and were stimulated under confluent conditions. The following chemicals were used for the experiments: BDNF (Peprotech), imipramine, amitriptyline, desipramine, chlorpromazine, phenelzine, clozapine, lithium (chloride salt). After the treatments, the medium was discarded and the cells lyzed in NP++ buffer and processed for western blot analysis or for phospho-Trk ELISA described below.

Phospho-Trk ELISA - An enzyme-linked immunosorbent assay (ELISA) method was developed to easily measure the level of phosphorylated Trk receptors from cultivated cells (**Figure S4A**). Whereas in Trk expressing cells the assay readily detects BDNF- or NGF-induced Trk phosphorylation, such induction is not detected in cells not expressing Trk receptors (data not shown). Moreover, BDNF-induced TrkB phosphorylation is lost if the cells are pretreated with Trk kinase inhibitor k252a (data not shown). Stimulation experiments were initiated by adding the drugs at different concentrations onto confluent cell line cultures or with primary neurons. After indicated incubation period at +37°C, the medium was removed and the cells were lyzed with cold NP++ buffer (50–100 µl). In a set of experiments, these steps were carried out by using Biomek FX workstation (Beckman Coulter) for the liquid handling and incubation. Following >30 min incubation on ice all material were transferred to pre-coated (sc-11-R, 1∶500, Santa Cruz Biotechnology; O/N at +4°C) and pre-blocked (2% BSA/PBS-T; 2 h at RT) white 96-well Optiplate™ (PerkinElmer) plates and 2–3% BSA/PBS-T (+2 mM Na_3_VO_4_) added *ad* 200 µl. The plates were incubated O/N at +4°C and thereafter the wells washed with PBS-T (4×300 µl) and anti-phosphotyrosine antibody added to the wells (4G10, Upstate; 1∶1000 in 5% NFDM/PBS-T or in house biotinylated PY20, AbD Serotec, 1∶1000 in 2% BSA/PBS-T; both O/N at +4°C). Following sequential washes and HRP-coupled tertiary antibody incubations (sheep anti-mouse-HRP, 1∶5000 in 5% NFDM/PBS-T or Streptavidin-HRP, 1∶10000 in 2% BSA/PBS-T; O/N at +4°C) 200 µl of ECL substrate (Pierce) was added to the wells and luminescence measured after 5 min with Varioskan Flash (Thermo Fisher Scientific) plate reader.

Western blotting and sugar digestions – Lectin precipitation was carried essentially as previously described [15] using *triticum vulgaris* (Amersham or EY Laboratories). A set of lectin precipitated samples were incubated with endoglycosidase-A (Endo-H) according to manufacturer's instructions (New England Biolabs). Proteins were separated in a SDS-PAGE under reducing conditions and blotted onto polyvinylidene difluoride (PVDF; Amersham) membrane. After blocking (3% BSA/TBST, 1 h, RT), the membranes were incubated with primary antibodies: anti-pY705/6 (own 1∶500–1000, [49]; Cell Signaling 1∶1000), anti-pY816 (1∶5000, a kind gift from Dr. Moses Chao, Skirball Institute, NY, USA), sc-11-R (1∶2000, Santa Cruz Biotechnology), anti-TrkB (1∶2000, BD Biosciences) or anti-BDNF (N-20/sc-546, Santa Cruz Biotechnology). Moreover, the membranes were washed with TBST and incubated with HRP-conjugated secondary antibody (1∶10000 in NFDM/TBST, 1 h, RT, Biorad). After subsequent washes, the secondary antibodies were visualized using ECL kits (Amersham Biosciences) followed by exposure to an X-ray film or Fuji LAS-3000 camera (Tamro Medlabs, Finland) for ECL detection.

Data quantitation and statistical analysis - Immunoblots were quantitated using NIH ImageJ 1.32. Statistical analyses were done using two-sample two-tailed Student t-test or when appropriate with one or two-way ANOVA followed with Newmann-Keuls *post hoc* test. Statistically significant *P*-value was set to 0.05. Data are presented as mean ± standard error of mean and as percentage of respective control.

## Supporting Information

Figure S1 **Diverse antidepressant drugs induce ∼105 kDa protein phosphorylation in the mouse brain.** **A**) Representative blot showing the time-response (30 min, 60 min, 120 min) of fluoxetine-induced (20/30 mg/kg, i.p.) phosphorylation of TrkB and ∼105 kDa protein (Y816 in left; Y705/6 in right) in mouse hippocampus. **B**) Representative blots showing antidepressant-induced phosphorylation of ∼105 kDa in mouse hippocampus **C**) Representative blots showing imipramine-induced phosphorylation of ∼105 kDa in mouse striatum, midbrain and whole brain homogenate. Abbreviations: FLX = fluoxetine, SAL = saline; CIT = citalopram; AMI = amitriptyline; CLO = clomipramine; MOC = moclobemide; REB = reboxetine; MW = molecular weight; TrkB.extr = antibody directed against the extracellular portion of TrkB receptors.
(TIF)

Figure S2 **Acute fluoxetine treatment did not regulate BDNF protein levels or tPA (tissue plasminogen activator) activity.** **A**) Representative blot showing mature-BDNF specific band in western blot from brain homogenates and pro- and mature-BDNF specific bands from respective control lanes as detected with polyclonal BDNF antibody (N-20/sc-546; Santa Cruz). Acute fluoxetine did not regulate mature-BDNF levels in mouse hippocampus. n = 6/group. **B**) Representative zymography showing caseinolysis at the level of recombinant tPA. Acute fluoxetine did not regulate tPA activity levels in mouse hippocampus. n = 6/group. Data is presented as percentage of control ± standard error of mean (SEM).
(TIF)

Figure S3 **Blockade of adenosine-A_2A_ receptor signaling does not prevent antidepressant-induced TrkB activation.** Acute imipramine treatment (30 mg/kg, i.p., 30 min; n = 6/group) induces essentially similar changes on TrkB phosphorylation in vehicle and adenosine-A_2A_ receptor antagonist (ZM241358; 1 mg/kg, i.p., 30 min) pre-treated mice. A representative blot in left showing imipramine-induced phosphorylation of TrkB and ∼105 kDa protein in mouse brain. Data is presented as percentage of control/saline ± standard error of mean (SEM). *<0.05; two-way ANOVA with Newmann-Keuls *post hoc* test.
(TIF)

Figure S4 **Phospho-TrkB enzyme-linked immunosorbent assay (ELISA).** **A**) Dose-response of BDNF (2, 8, 20 ng/ml, 15 min) on TrkB phosphorylation in TrkB expressing fibroblasts cultivated in 48-well plates. n = 4/group. **B**) Whereas BDNF produces robust TrkB phosphorylation in TrkB expressing fibroblasts cultivated in 24-well plates, all the tested drugs at selected doses did not have any effect on TrkB phosphorylation (compounds incubated for 15 min). n = 3/group. Data is presented as percentage of control ± standard error of mean (SEM). Abbreviations: IMI = imipramine; PHE = phenelzine; FLX = fluoxetine; CLOZ = clozapine; Li = lithium chloride; CHLOR = chlorpromazine.
(TIF)
