# Supplementary figures and images for: Antidepressant Drugs Transactivate TrkB Neurotrophin Receptors in the Adult Rodent Brain Independently of BDNF and Monoamine Transporter Blockade
Source: PLoS One. 2011 Jun 7;6(6):e20567. doi: 10.1371/journal.pone.0020567 (PMC3110188; doi:10.1371/journal.pone.0020567)

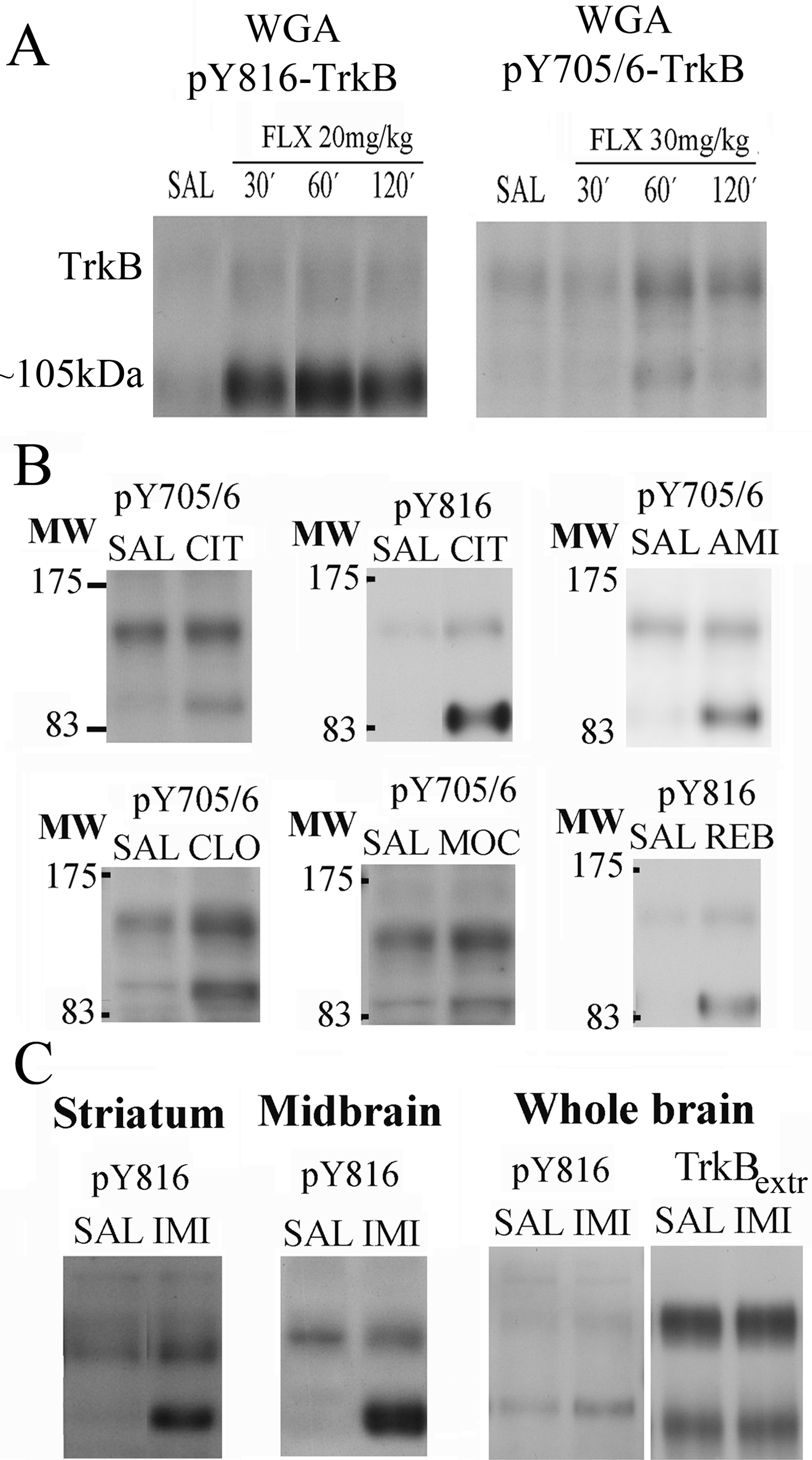

Supplement: Figure S1 — Diverse antidepressant drugs induce ∼105 kDa protein phosphorylation in the mouse brain. A) Representative blot showing the time-response (30 min, 60 min, 120 min) of fluoxetine-induced (20/30 mg/kg, i.p.) phosphorylation of TrkB and ∼105 kDa protein (Y816 in left; Y705/6 in right) in mouse hippocampus. B) Representative blots showing antidepressant-induced phosphorylation of ∼105 kDa in mouse hippocampus C) Representative blots showing imipramine-induced phosphorylation of ∼105 kDa in mouse striatum, midbrain and whole brain homogenate. Abbreviations: FLX = fluoxetine, SAL = saline; CIT = citalopram; AMI = amitriptyline; CLO = clomipramine; MOC = moclobemide; REB = reboxetine; MW = molecular weight; TrkB.extr = antibody directed against the extracellular portion of TrkB receptors. (TIF) [file pone.0020567.s001.tif]

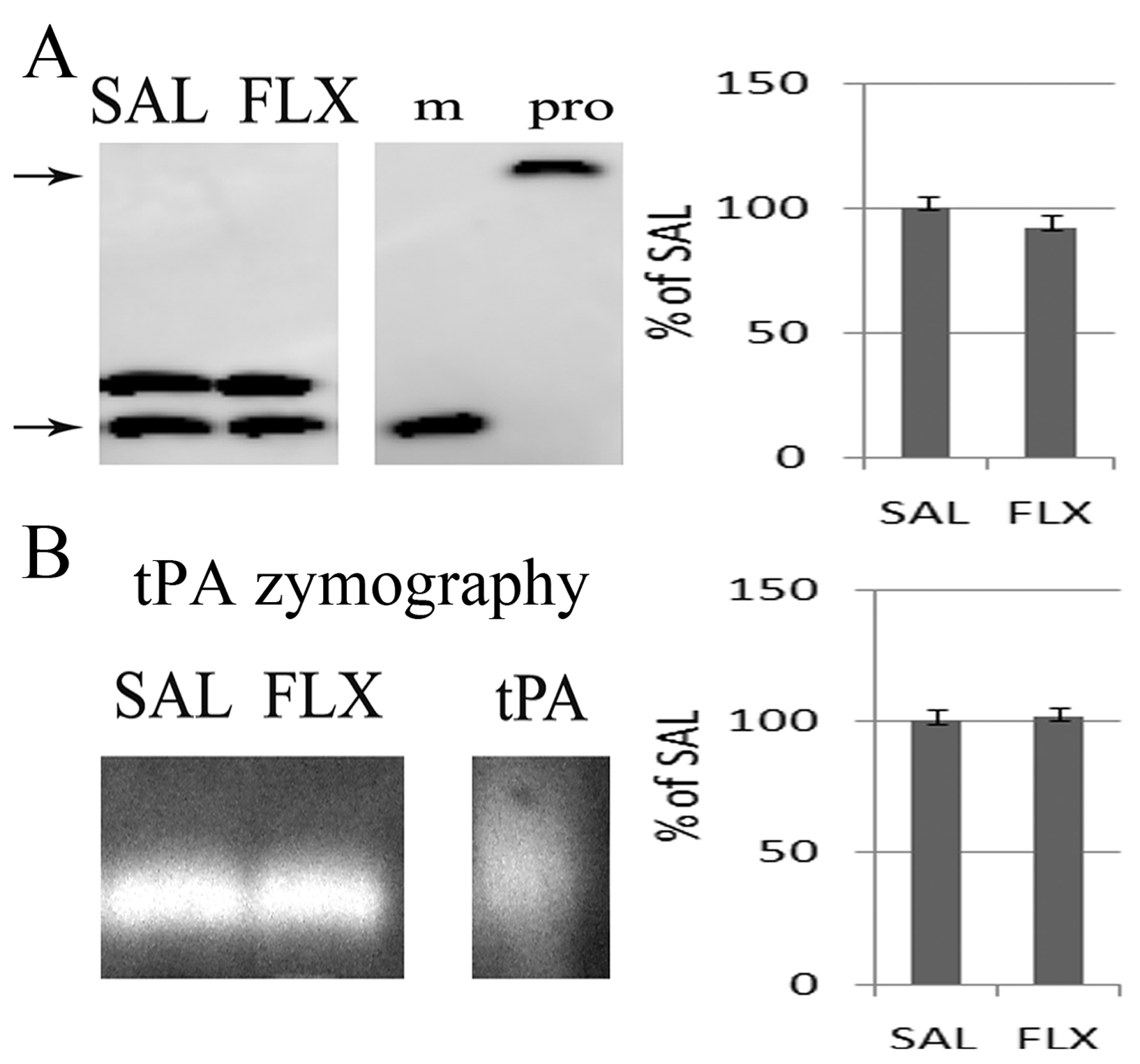

Supplement: Figure S2 — Acute fluoxetine treatment did not regulate BDNF protein levels or tPA (tissue plasminogen activator) activity. A) Representative blot showing mature-BDNF specific band in western blot from brain homogenates and pro- and mature-BDNF specific bands from respective control lanes as detected with polyclonal BDNF antibody (N-20/sc-546; Santa Cruz). Acute fluoxetine did not regulate mature-BDNF levels in mouse hippocampus. n = 6/group. B) Representative zymography showing caseinolysis at the level of recombinant tPA. Acute fluoxetine did not regulate tPA activity levels in mouse hippocampus. n = 6/group. Data is presented as percentage of control ± standard error of mean (SEM). (TIF) [file pone.0020567.s002.tif]

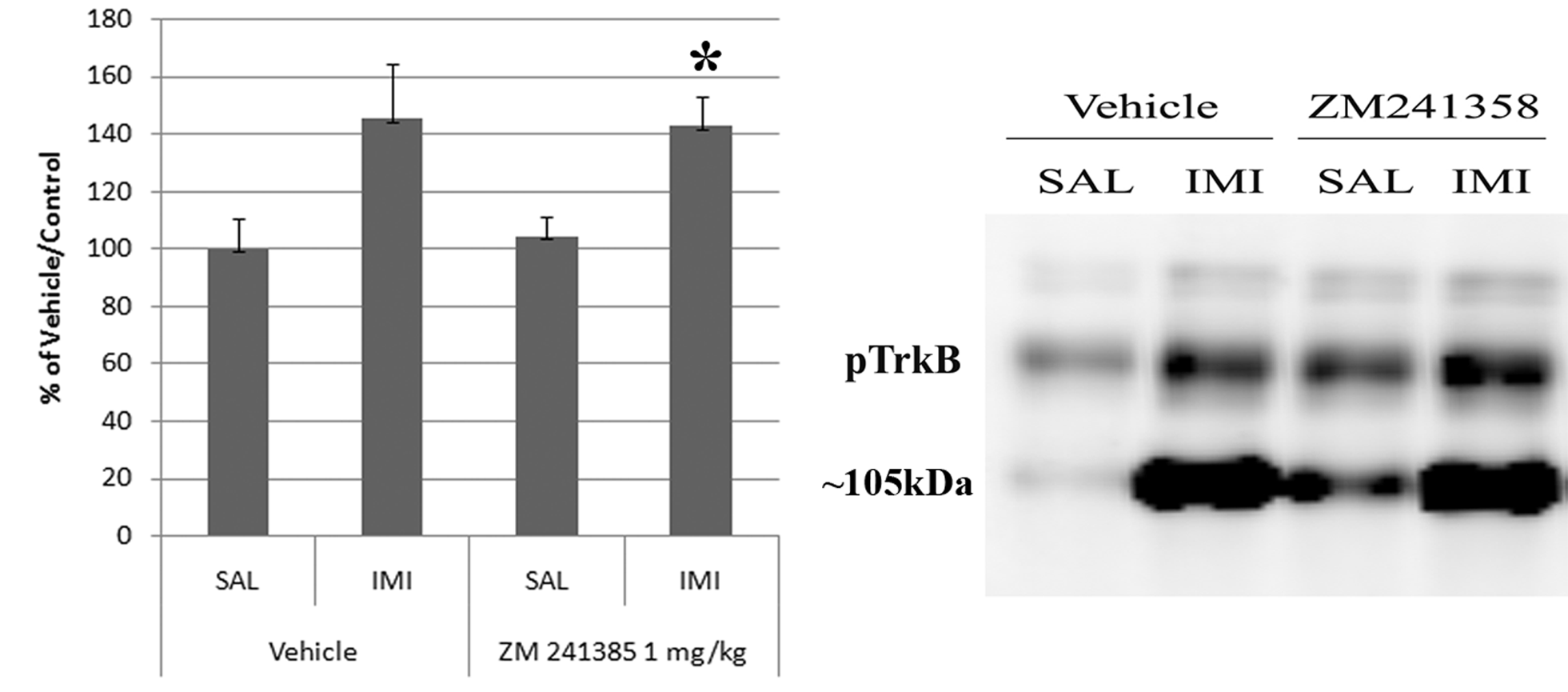

Supplement: Figure S3 — Blockade of adenosine-A2A receptor signaling does not prevent antidepressant-induced TrkB activation. Acute imipramine treatment (30 mg/kg, i.p., 30 min; n = 6/group) induces essentially similar changes on TrkB phosphorylation in vehicle and adenosine-A2A receptor antagonist (ZM241358; 1 mg/kg, i.p., 30 min) pre-treated mice. A representative blot in left showing imipramine-induced phosphorylation of TrkB and ∼105 kDa protein in mouse brain. Data is presented as percentage of control/saline ± standard error of mean (SEM). *<0.05; two-way ANOVA with Newmann-Keuls post hoc test. (TIF) [file pone.0020567.s003.tif]

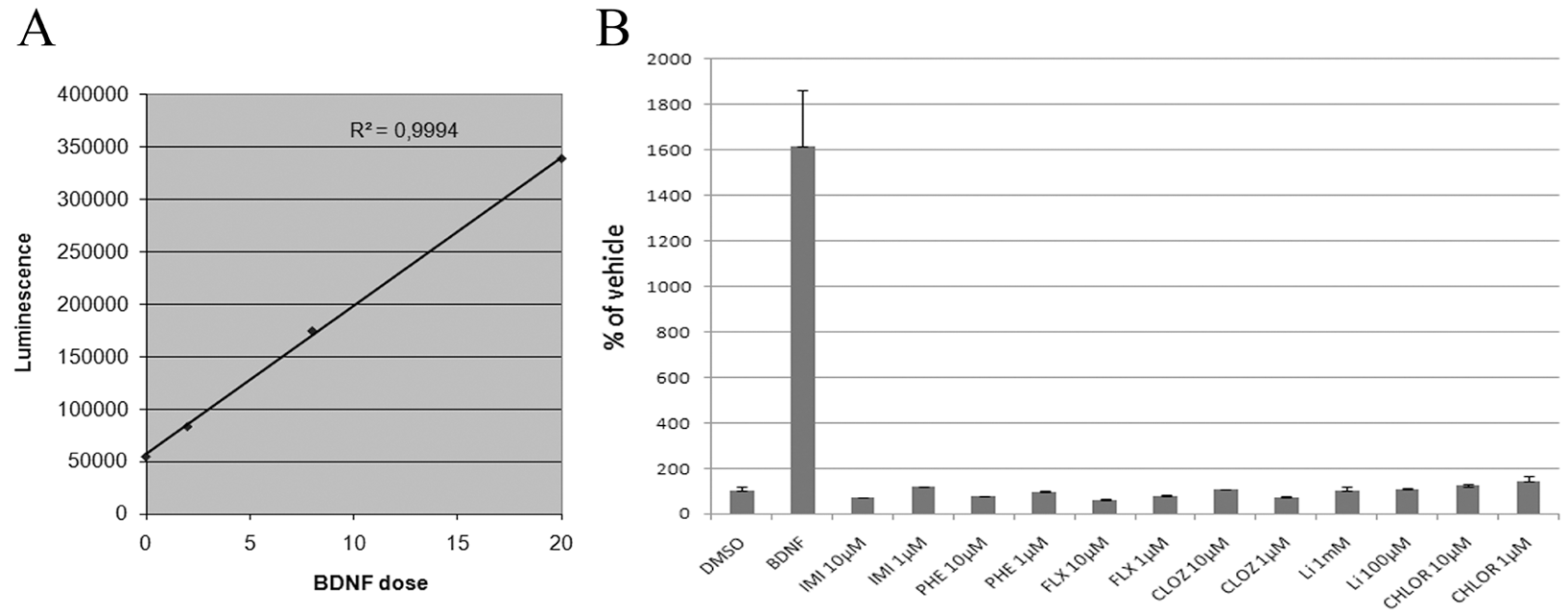

Supplement: Figure S4 — Phospho-TrkB enzyme-linked immunosorbent assay (ELISA). A) Dose-response of BDNF (2, 8, 20 ng/ml, 15 min) on TrkB phosphorylation in TrkB expressing fibroblasts cultivated in 48-well plates. n = 4/group. B) Whereas BDNF produces robust TrkB phosphorylation in TrkB expressing fibroblasts cultivated in 24-well plates, all the tested drugs at selected doses did not have any effect on TrkB phosphorylation (compounds incubated for 15 min). n = 3/group. Data is presented as percentage of control ± standard error of mean (SEM). Abbreviations: IMI = imipramine; PHE = phenelzine; FLX = fluoxetine; CLOZ = clozapine; Li = lithium chloride; CHLOR = chlorpromazine. (TIF) [file pone.0020567.s004.tif]
